# Construction and immunogenicity of a recombinant swinepox virus expressing a multi-epitope peptide for porcine reproductive and respiratory syndrome virus

**DOI:** 10.1038/srep43990

**Published:** 2017-03-08

**Authors:** Huixing Lin, Zhe Ma, Xin Hou, Lei Chen, Hongjie Fan

**Affiliations:** 1 College of Veterinary Medicine, Nanjing Agricultural University, Nanjing 210095, China; 2 Jiangsu Co-innovation Center for Prevention and Control of Important Animal Infectious Diseases and Zoonoses, Yangzhou, China

## Abstract

To characterize neutralizing mimotopes, phages were selected from a 12-mer phage display library using three anti-porcine reproductive and respiratory syndrome virus (PRRSV) neutralizing monoclonal antibodies: (1) A1; (2) A2; and (3) A7. Of these, A2 and A7 recognize the mimotope, P2, which contains the SRHDHIH motif, which has conserved consensus sequences from amino acid positions 156 to 161 in the N-terminal ectodomain of GP3. The artificial multi-epitope gene, *mp2*, was designed by combining three repeats of the mimotope P2. The resulting sequence was inserted into the swinepox virus (SPV) genome to construct a recombinant swinepox virus (rSPV-mp2). The rSPV-mp2 was able to stably express the multi-epitope peptide, mP2, *in vitro*. The rSPV-mp2 immunized pigs exhibited a significantly shorter fever duration compared with the wtSPV treated group (*P* < 0.05). There was an enhanced humoral and cellular immune response, decreased number of PRRSV genomic copies, and a significant reduction in the gross lung pathology (*P* < 0.05) was observed following PRRSV infection in rSPV-mp2-immunized animals. The results suggest that the recombinant rSPV-mp2 provided pigs with significant protection against PRRSV infection.

Porcine reproductive and respiratory syndrome (PRRS) is an economically costly disease characterized by reproductive failure in sows, and respiratory disease in pigs^1^,^2^. The PRRS epidemic is caused by the highly pathogenic porcine reproductive and respiratory syndrome virus (HP-PRRSV), and has resulted in huge economic losses to the Chinese pig industry since 2006^3^,^4^,^5^,^6^,^7^. Neutralizing antibodies (NA) are capable of blocking PRRSV infections by preventing the virus from binding to the sialoadhesin (Sn) receptor on macrophages^8^,^9^. The role of NA in protection against PRRSV infection was demonstrated in pigs by passive antibody transfer^10^. Several viral targets that play a role in the induction of NA have been reported, including GP3, GP4, GP5, and M proteins^11^,^12^,^13^,^14^.

To control PRRS, vaccines derived from inactivated or attenuated PRRSV strains are commercially available^15^. However, the efficacy of the current PRRSV vaccines, has proven less than satisfactory, because the attenuated vaccines may not be completely safe and could not provide sufficient protection against heterologous infection^16^,^17^, and the inactivated vaccines require frequent injections over a long period of time to be effective^18^,^19^, emphasizing the need for newer, more efficient vaccines. Recently, significant progress has been made regarding the development of new vaccination strategies against PRRSV^20^,^21^. Recombinant viral genome vaccines against PRRSV may represent an alternative for the generation of live, attenuated viral vaccines^22^. The swinepox virus (SPV) is a promising vaccine vector due to its ability to stimulate the immune response, and its capacity for heterogeneous insertions^23^,^24^. Moreover, SPV has the additional advantage of a low production cost, ease of administration, and strict host range, providing the potential to be an effective vaccine carrier for wide use in the expression of exogenous genes^25^,^26^. Epitope-based vaccines (EVs) are another alternative^27^, as the immune response evoked towards the EV is directed only towards the relevant epitopes of the pathogen, and may avoid the pathogenic effects associated with the administration of integral antigens^28^. Random peptide libraries (RPLs) have been used to identify relevant epitopes on protein antigens^29^. Peptide mimics of linear and discontinuous protein epitopes, as well as other non-protein antigens, can be affinity purified from RPLs^30^. The peptide epitope mimics (i.e., mimotopes) used as immunogens are a promising tool for vaccine design, and an efficient means of stimulating the antibody response to native antigens, and providing protection against infection *in vivo*^31^.

In the present study, a recombinant swinepox virus (rSPV-mp2) expressing the multi-epitope peptide for PRRSV was constructed. The replication and expression of the rSPV-mp2 in PK-15 cells were characterized, and the immunogenicity of the rSPV-mp2 was investigated in pigs. Our novel construct may have potential as a porcine-based vaccine against PRRSV infection.

## Results

### Enrichment of specific phage peptides by mAbs

The peptides recognized by the neutralizing mAbs A1, A2 and A7 were screened from a 12-mer phage display library. The neutralizing mAbs, A2 and A7, were both targeted PRRSV GP3, and the target antigen of the neutralizing mAb, A1 was not clear. The number of recovered phages after the third panning was at least 100-fold higher than that after the first panning in all of the selections performed (Table 1). A more than 500-fold increase was observed for mAb A2, while the fold enrichment using mAbs A1 and A7 was 167 and 138, respectively.

### Specificity analysis of positive phage clones

An ELISA was used to confirm that the phage-displayed peptides were specifically recognized by mAbs; PRRSV-negative serum was used as a negative control. To test the binding specificity, 150 phage colonies (50 for each mAb) were randomly selected. For each mAb, the first 12 clones with higher A_450_ values were chosen as the positive phage clones to perform a competitive inhibition ELISA. The results revealed an inhibition level of 39.2%, 35.7%, and 35.0% for mAbs A1, A2, and A7, respectively.

### Sequence determination of the phage-display peptides

**Figure 1:**
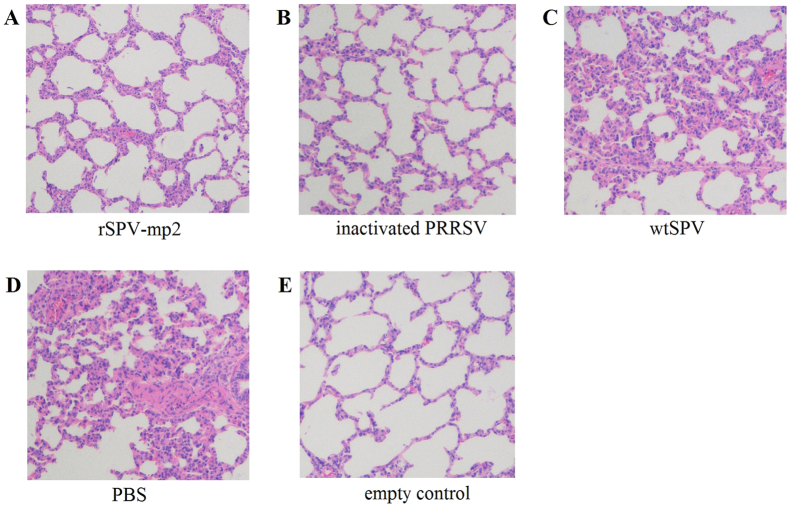
Alignment of the peptide sequences using ClustalW. (**A**) Dodecapeptide sequences of the phages selected using three neutralizing mAbs. (**B**) Homologous alignment of the primary sequence of GP3 to the P2 mimotope. Two representative sequences of mimotopes corresponding to mAbs were determined.

Sequencing of all the selected positive phage clones revealed the correct library insert sequence (LIS). Consensus peptide sequences were identified from the third round of biopanning (Fig. 1A). The P1 mimotope recognized by neutralizing MAb A1 contained the NWKN motif, which was found to have no significant consensus sequences for any of the PRRSV proteins. Another mimotope referred to as P2, was recognized by neutralizing MAb A2 and A7. The SRHDHIH motif in mimotope P2 was found from the amino acid positions 156 to 161 in the N-terminal ectodomain of GP3. However, no significant consensus sequences were found for P1 (Fig. 1B).

### Identification of recombinant viruses

**Figure 2:**
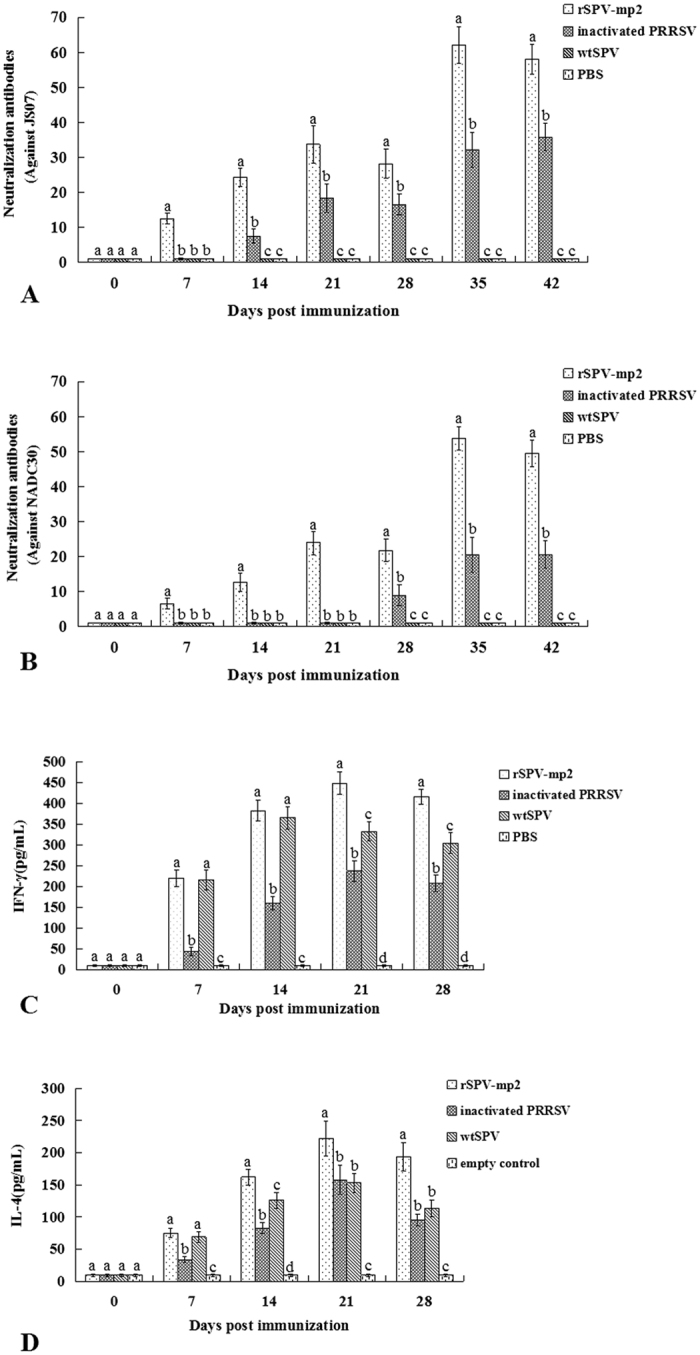
The design and identification of the recombinant virus. (**A**) The mP2 was constructed from three repeats of the P2 mimotope, with three glycine residues and a serine residue (GGGS) as a spacer peptide. (**B**) The construction of the pUSG11/P28-mp2 transfer vector. LF and RF respectively, indicate the SPV left and right flanking sequences, respectively. P11 and P28 are the vaccinia virus (VV) promoters. The GFP reporter gene is also included in the plasmid. (**C**) The recombinant virus rSPV-mp2 was constructed from the wtSPV homologous recombinant pUSG11/P28-mp2. (**D**) Western blot analysis of mP2 expression in PK-15 cells (uncropped): Lane 1: PK-15 cells infected with rSPV-mp2; Lane 2: PK-15 cells infected with wtSPV. (**E,F**) Indirect immunofluorescence assay of the rSPV-mp2. (**E**) Red fluorescence was observed in rSPV-mp2 infected PK15 cells, in which the fluorescence was localized to the cytoplasm. (**F**) No fluorescence was observed in PK15 cells infected with wtSPV.

A Western blot was carried out to verify the expression of mP2 in PK-15 cells infected with rSPV-mp2. We used an A2 mAb to probe for a protein of approximately 5.5 kDa in size, which was detected in rSPV-mp2 infected cells (Fig. 2D). An indirect immunofluorescence assay (IFA) was used to further verify the expression and localization of mP2 in PK-15 cells infected with rSPV-mp2. A strong red fluorescence signal was observed in the rSPV-mp2 infected cells, whereas no specific red fluorescence was detected in the wtSPV-infected cells (Fig. 2E and F). After more than 30 passages of rSPV-mp2, both the Western blot and IFA analysis identified the stable expression of mP2.

### Clinical evaluation

**Figure 3:**
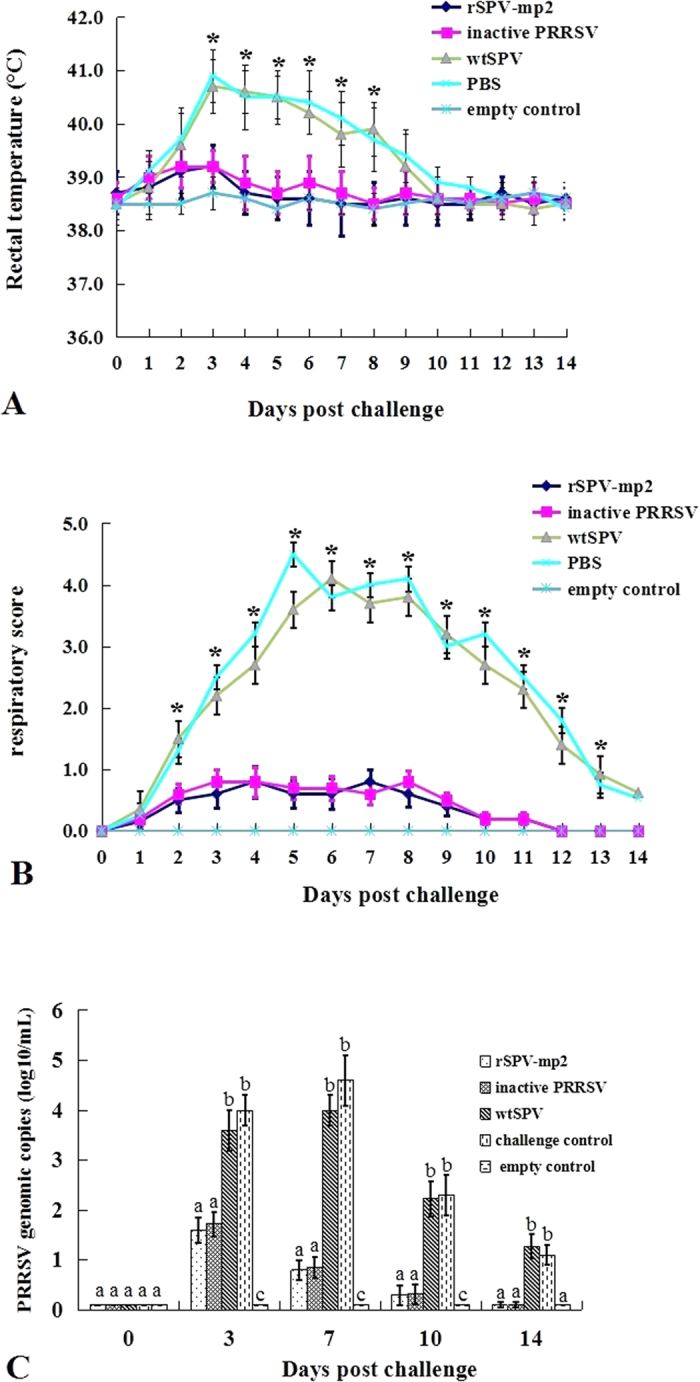
Clinical evaluation of pigs, and genomic copies of PRRSV in the serum samples from the different groups post-PRRSV challenge. (**A**) Mean rectal temperatures. (**B**) Mean respiratory scores, ranging from 0 (normal) to 6 (severe dyspnea and abdominal breathing). (**C**) Genomic copies of PRRSV in the serum samples post-challenge. *Indicates a significantly difference (*P* < 0.05) of the rSPV-mp2 immunized and the inactivated PRRSV immunized groups compared to the wtSPV treated and the PBS treated groups. Different letters (a, b, and c) indicate significant difference between the groups.

Prior to PRRSV challenge, no clinical signs were observed in any of the five groups. Post-PRRSV challenge, no pigs died in any of the five groups. All pigs from the wtSPV-treated group and PBS-treated group developed pyrexia, difficulty breathing, and growth retardation. The mean rectal temperatures of the pigs in the wtSPV treated group, and the PBS-treated group was significantly higher (*P* < 0.05) than that in the rSPV-mp2 immunized group and inactive PRRSV immunized group from 3 to 8 days post challenge (dpc; Fig. 3A). The mean respiratory scores of pigs in the wtSPV-treated and the PBS-treated group were significantly higher (*P* < 0.05) than the rSPV-mp2 immunized group and the inactive PRRSV-immunized group from 2 to 13 dpc (Fig. 3B). These results indicate that immunization with rSPV-mp2 was able to protect the pigs from clinical signs of PRRS following viral challenge.

### Quantification of PRRSV in the serum samples

PRRSV genomic copies were not detected in any of the serum samples at 0 dpc in any of the pigs. They were significantly increased by 7 dpc. The number of PRRSV genomic copies in the serum was significantly lower in the rSPV-mp2 immunized and the inactivated PRRSV-immunized groups compared with the wtSPV-treated and the PBS-treated groups for all time points post-challenge (*P* < 0.01). There were no PRRSV genomic copies detected in the serum samples of the empty control pigs throughout the experiment (Fig. 3C). This result indicated that immunization with rSPV-mp2 could reduce the viremia following viral challenge.

### The antibody response to rSPV-mp2 following vaccination

**Figure 4:**
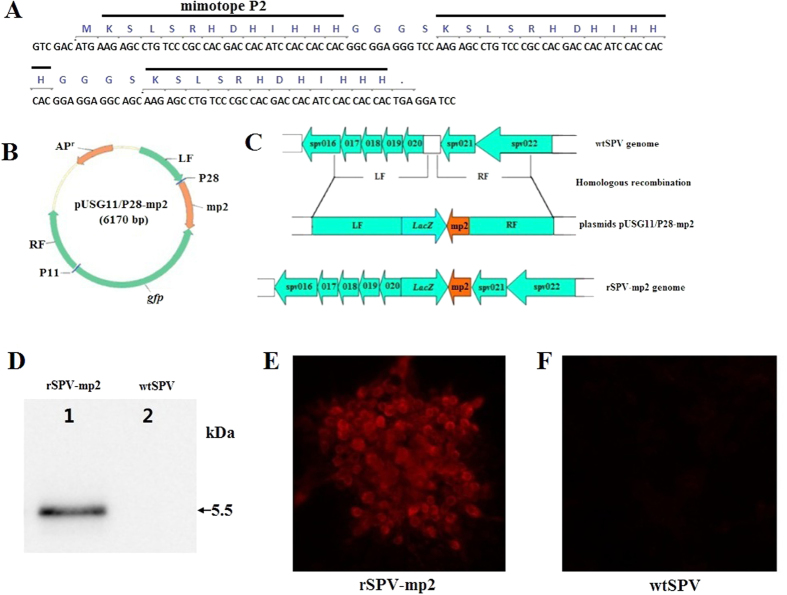
The neutralizing antibody response and the immune response to rSPV-mp2 following vaccination. (**A,B**) Mean values of the PRRSV-neutralizing antibody titres against homologous (JS07) and heterogeneous (NADC30) PRRSV strains of pigs in different groups post immunization. (**C,D**) The concentration of serum IFN-γ and IL-4 post immunization. Different letters (a, b, c and d) indicate a significant difference (*P* < 0.05) between the groups.

The antibody response elicited after immunization with rSPV-mp2 was monitored by determining the serum neutralizing antibody titres for all of the tested pigs. The titres of neutralizing antibodies against homologous and heterogeneous PRRSV strains in the pigs vaccinated with rSPV-mp2 increased following primary immunization, and were significantly higher (*P* < 0.05) than the inactivated PRRSV immunized group for all post-immunization time points (Fig. 4A,B). In the wtSPV-treated and PBS-treated groups, none of the neutralizing antibodies against PRRSV could be detected throughout the experiment.

### Detection of the immune response

As shown in Fig. 4, the rSPV-mp2-immunized group induced a significantly higher level of IFN-γ (Fig. 4C), as well as IL-4 (Fig. 4D), compared to the inactivated PRRSV immunized group (*P* < 0.05). It indicated that both the Th1-type and Th2-type of immune responses were enhanced by rSPV-mp2 in animals.

### Lung lesions

Gross lesions were observed in the wtSPV-treated, and the PBS-treated group, including varying degrees of red-to-purple consolidation in the lung tissues. A significant reduction in the gross lung lesion scores was noted in the rSPV-mp2 immunized group, compared with the wtSPV-treated and the PBS-treated groups (*P* < 0.01). However, no significant differences were observed between the rSPV-mp2-immunized and the inactivated PRRSV-immunized groups. No significant gross lesions were observed in the untreated control group (Table 2).

**Figure 5:**
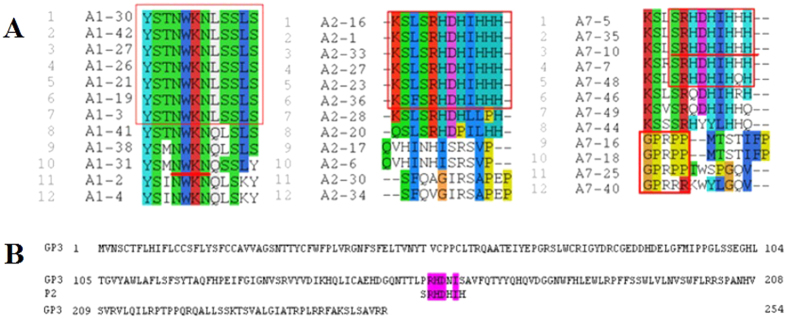
The morphometric analysis of the histopathological pulmonary changes.

In the morphometric analysis of the histopathological pulmonary changes, mildly thicker alveolar walls and macrophage infiltration of alveolar septa in the lung were observed in the wtSPV-treated and the PBS-treated groups (Fig. 5). A significant reduction in the histopathological lung lesion scores was noted in the rSPV-mp2-immunized group compared with the wtSPV-treated and the PBS-treated groups (*P* < 0.01, Table 2). In addition, no significant difference was observed between the wtSPV-treated and the PBS-treated group. No histopathological pulmonary lesions were observed in the empty control group (Table 2).

### Immunohistochemistry

The lung tissues were collected for immunohistochemistry (IHC) at the time of necropsy, as previous described^32^. The mean number of PRRSV-positive cells per unit area of the lung tissues from the pigs in the wtSPV-treated group was significantly higher than that of the rSPV-mp2-treated and the inactive PRRSV-immunized groups (*P* < 0.01; Table 2).

## Discussion

GP3 is one of the essential envelope proteins of PRRSV, and there is limited information regarding its antigenic structure^33^. Nelson *et al*. hypothesized that there was a conformational epitope embodied in GP3, but it was never identified^34^. In this study, one PRRSV-specific mimotope recognized by an anti-PRRSV neutralizing mAb, A2, was identified from a phage-displayed 12-mer peptide library. The mimotope contains the SRHDHIH motif, which was found at the amino acid positions 156 to 161 in the N-terminal ectodomain of GP3. Moreover, epitope prediction revealed that there were high levels of immunogenicity and hydrophilicity in this region, which was likely to be a linear neutralizing epitope^35^. Our findings will provide a basis for determining the antigenic structure of GP3.

Previous studies have demonstrated that PRRSV-neutralizing antibodies play a critical role in the clearance of the virus, and can protect pigs against PRRSV infection^36^. An ideal PRRSV vaccine should be able to induce a more rapid and robust neutralizing antibody response following infection with PRRSV. Experimental subunit vaccines based on different viral vector systems that express several PRRSV antigens, such as GP5, M, and GP3, have been developed and evaluated for their effect against PRRSV^21^,^37^. Previous studies showed that the anti-PRRSV neutralizing antibodies were detectable until 28 dpi or later^37^. In this study, the neutralizing antibodies against homologous strain of group 2 (inactivated PRRSV-JS07 with adjuvant) was detectable within 14–21 dpi, and the neutralizing antibodies against heterologous strain of group 2 was detectable until 28 dpi. That maybe because the homologous strain was more sensitive to the neutralizing antibodies than the heterologous strain. At 7 dpi, the pigs in the rSPV-mp2 immunized group produced a significant PRRSV-specific neutralizing antibodies with titers of 1:16, while no PRRSV-specific neutralizing antibodies were detected in the inactivated PRRSV-immunized group. At all post-immunization time points tested, the PRRSV-specific neutralizing antibody titers of the rSPV-mp2 immunized group were significantly higher than that of the inactivated PRRSV-immunized group (*P* < 0.05). The results indicated that the SPV vector could apply as an adjuvant, that enhance both the cellular immunity and the humoral immunity. Immunization with rSPV-mp2 could induce more rapid and robust PRRSV-specific neutralizing antibodies, and provide pigs with significant protection against PRRSV infection.

The complexity of the immune response to PRRSV, and the ability of the virus to escape or modulate the host’s immune system make it difficult to develop a vaccine that can be used to eradicate the disease^1^,^38^. However, rational design is an important factor influencing vaccine efficacy. In the present study, we constructed an SPV-based recombinant virus, (rSPV-mp2) that expressed a multi-epitope peptide for PRRSV. The findings of our study demonstrate that rSPV-mp2 could protect pigs from viremia and clinical signs of PRRS following viral challenge, and is capable of inducing enhanced humoral and cellular immune responses in pigs. Therefore, it may serve as a promising candidate vaccine against PRRSV infection. Before this vaccine can be applied, further studies are required to determine if this vaccine confers more efficient protection than existing vaccines targeted against various strains of PRRSV.

## Materials and Methods

### Viruses and animals

In Jiangsu province, a highly pathogenic PRRSV (strain JS07) was isolated from the lungs of pigs in 2007, and designated as the North American genotype^39^. The SPV (VR-363) was purchased from the American Type Culture Collection (ATCC). Specific pathogen free four-week-old female BALB/c mice, were purchased from the Comparative Medicine Center of Yangzhou University. Specific pathogen free Bama minipigs were purchased from Shanghai Academy of Agricultural Sciences. All experimental protocols were approved by the Laboratory Animal Ethical Committee of Nanjing Agricultural University and performed accordingly.

### Preparation of neutralizing monoclonal antibodies

PRRSV was propagated in Marc-145 cells and purified using a sucrose density gradient separation. Briefly, the virus was released by three rounds of freezing and thawing of the cell cultures, and then was concentrated from the supernatant via ultracentrifugation at 120,000 × g for 2 h at 4 °C. The viral pellet was then resuspended in phosphate buffered saline (PBS), layered on 30 and 60% (w/v) sucrose gradients, and centrifuged at 100,000 × g for 2 h and 4 °C. The purified virus band was collected and resuspended in PBS, and centrifuged at 120,000 × g for 2 h at 4 °C to pellet the purified virus. Finally, the purified-PRRSV antigen was resuspended in PBS and stored at −80 °C. BALB/c mice (n = 10) were intraperitoneally injected twice (on day 1 and 14) with 20 μg of purified PRRSV antigens (JS07). The first injection was in complete Freund’s adjuvant (Sigma-Aldrich) and the second was in incomplete Freund’s adjuvant (Sigma-Aldrich). Two weeks after the second immunization, the mice were intraperitoneally injected with 5 μg of purified PRRSV antigen (prepared in our laboratory) in PBS. The spleens from the mice were collected three days after the boost was administered, and was used for fusion with SP2/0 myeloma cells following the standard polyethyleneglycol-mediated cell fusion procedure^40^. Hybridomas secreting antibodies were screened by an in-house PRRSV-specific indirect ELISA, and those that were PRRSV-positive were cloned using a limiting dilution approach^41^. The ability of monoclonal antibodies (mAbs) to neutralize the infectivity of PRRSV was assessed in microtiter trays as described previously^42^. Briefly, two-fold serially diluted mAbs (50 μL) were mixed with an equal volume of 200 TCID_50_ of the PRRSV strain JS07 or NADC30 strains (provide by the Jiangsu Academy of Agricultural Sciences), respectively, in 96-well culture plates and incubated for 1 h at 37 °C and 5% CO_2_. Following the incubation, 100 μL Marc-145 cell suspension containing 2 × 10^4^ cells was added to each well. The plates were incubated at 37 °C in a humidified atmosphere containing 5% CO_2_ and examined daily for up to six days for the appearance of a PRRSV-specific cytopathic effect (CPE). The neutralization titers were expressed as the reciprocal of the highest serum dilution in which no CPE was observed. We obtained three mAbs which exhibited a strong neutralizing ability against PRRSV JS07 strain (Table 3). The mAbs in the ascitic fluids were purified by saturated ammonium sulphate precipitation and Protein-G chromatography (GE Healthcare).

### Identification of mimotopes from random peptide libraries

A Ph.D.-12 Phage Display Peptide Library with a concentration of 1.5 × 10^13^ PFU/mL and complexity of 2.7 × 10^9^ transformants was purchased from New England Biolabs. Three rounds of biopanning were carried out following the manufacturer’s instructions. For rapid screening of positive phage clones, sandwich ELISAs, and competitive inhibition ELISAs were carried out to analyse specificity as described previously^43^. Phage-containing supernatants from a single positive clone were collected for purification of sequencing templates^44^. After the single-stranded phage DNA was sequenced, the amino acid sequences were deduced, and the peptide sequences were identified. Consensus peptide sequences were identified by sequence alignment using ClustalW (http://www.ebi.ac.uk/clustalw/)^45^.

### Construction of the transfer vector

A multi-epitope fragment derived from the PRRSV strain JS07 (i.e., mp2) was found to contain three repeats of mimotope P2 linked by four 4-peptide (GGGS) spacers between neighbouring mimotopes as shown in Fig. 2A. The entire *mp2* gene was synthesized and cloned into the pGH-T vector to generate the pGH-mp2 plasmid. Previously, we have developed a vector system based on the pUSG11/P28 plasmid for the construction of recombinant SPV vectors carrying foreign genes^23^. The *mp2* gene was amplified from the pGH-mp2 plasmid using primers mp2F (5′-CTCGAGATGAAGAGCCTGTCCCGCC-3′) and mp2R (5′-GGATCCTTATTAGTGGTGGTGGATGTGGTCG-3′), and then inserted into the Sal I-BamH I multiple cloning sites of the pUSG11/P28 plasmid to create the transfer vector, pUSG11/P28-mp2 (Fig. 2B).

### Generation and identification of recombinant viruses

The recombinant swinepox virus rSPV-mp2 was constructed by wtSPV homologous recombination with pUSG11/P28-mp2 (Fig. 2C). Briefly, PK-15 cells were routinely cultured at 37 °C and 5% CO_2_ in Eagle’s Minimum Essential Medium (EMEM), supplemented with 8% fetal bovine serum (FBS). PK-15 cells were grown in a six-well plate, and infected with SPV (m.o.i. of 0.05) for 1 h, and subsequently transfected with 4 μg of the pUSG11/P28-mp2 plasmid using ExFect Transfection Reagent (Vazyme Biotech Co., Ltd., China). The cells were collected after four days of incubation at 37 °C and 5% CO_2_ in 2 mL EMEM medium with 1% FBS. The recombinant rSPV-mp2 virus was purified and identified as previous described^25^.

### Animal experimental design

A total of 25 four-week-old specific pathogen free Bama minipigs were randomly divided into five groups (n = 5 per group). At 28 days of age (0 days post-immunization [dpi]), pigs in group 1 were immunized intramuscularly with 2 × 10^6^ PFU of rSPV-mp2 (2 mL); group 2 was immunized with 2 × 10^5^ TCID_50_ (1 mL) inactivated PRRSV-JS07 mixed with an equal amount of ISA201 adjuvant (SEPPIC, France) as positive controls; group 3 was immunized with 2 × 10^6^ PFU of wild-type SPV (wtSPV, 2 mL) as the negative control; group 4 was treated with 2 mL of PBS as challenge control; and group 5 remained untreated as empty control.

At 56 days of age (0 days post-challenge [dpc]), all the pigs in groups 1–4 were challenged intranasally with 5 mL (1.2 × 10^5^ TCID50/mL) of PRRSV-JS07; and group 5 was left unchallenged as an empty control. The challenge was slowly dripped into both nostrils of the pigs, taking approximately 5 min/pig.

### Clinical observations

Following vaccination and challenge, the pigs were monitored daily for their physical condition, and the clinical respiratory disease severity was scored on a scale from 0 (normal) to 6 (severe dyspnoea and abdominal breathing)^46^. Rectal temperatures were also recorded daily.

### Quantification of PRRSV in the blood

Serum samples were collected from all pigs at 0, 3, 7, 10, and 14 dpc. Genomic PRRSV copies were detected by real-time PCR as previously described^47^. A pair of primers:

MF: 5′ CAGATGCCGTTTGTGCTTGTTAGG-3′;

MR: 5′ CACCAATGTGCCGTTGACCGTAG-3′

was designed with specificity to the PRRSV genome. A 154 bp gene fragment was amplified by PCR from the PRRSV genomic RNA using this pair of primers, and then inserted into the Simple 19 T vector (Takara) to construct the 19T-M plasmid. The plasmid 19T-M was transformed into a competent *E. coli* DH5α, and the transformants were subsequently selected using a selection plate containing ampicillin. The ampicillin positive clones were confirmed by PCR and sequencing with a pair of primers (BcaBEST Sequencing Primer RV-M/BcaBEST Sequencing Primer M13-47) on the flanking region of the inserted sequence. After confirmation by PCR and sequencing, plasmid 19T-M was transformed into competent *E. coli* DH5α to produce large amounts of 19T-M plasmid. Serial dilutions of the 19T-M plasmid were used to establish the standard curve for real-time PCR. The sensitivity and the specificity of the real-time PCR were then determined (data not shown).

Viral RNA was extracted from the serum samples using the Viral RNA Mini Kit (QIAGEN) according to the manufacturer’s instructions. The amplification was performed with 50 pmol of each primer, 25 μL of 2 × SYBR green quantitative PCR master mix (TaKaRa), 5 μL of the extracted RNA, and nuclease free water to 50 μL. The reaction was run in the 7300/7500 Real-Time PCR System (Applied Biosystems) with the following program: 10 s at 95 °C, followed by 40 cycles of 5 s at 95 °C, 30 s at 55 °C. The internal control was cloned from the PRRSV genomic RNA, and revealed a 154 bp fragment with the same primers. Briefly, the crude stock of PRRSV was prepared by infecting the cultured Marc-145 cells for three days at 37 °C, followed by freezing and thawing the cells three times. The viral genomic RNA was extracted from the crude stock of PRRSV using the Viral RNA Mini Kit (QIAGEN) according to the manufacturer’s instructions. The genomic PRRSV RNA was used as a template for the real-time PCR internal control. Two independent tests were conducted in triplicate.

### Serology

A serum neutralization assay was performed to determine the titre of the PRRSV-neutralizing antibodies in the serum samples. Sera from all animals in each immunization group was collected at 0, 7, 14, 21, 28, 35, and 42 dpi. The samples were heat-inactivated for 30 min at 56 °C. Two-fold serial dilutions of the test sera, starting at 1:10, were incubated for 60 min at 37 °C in the presence of 200 TCID_50_ of PRRSV-JS07 or NADC30 strains (provide by the Jiangsu Academy of Agricultural Sciences), respectively, in DMEM medium. The mixtures were added to 96-well microtitration plates containing confluent MARC-145 cells seeded 12 h prior to the experiment. After incubation for four days at 37 °C in a humidified atmosphere containing 5% CO_2_, cells were examined for cytopathic effects (CPE). The titres of the neutralizing antibodies towards PRRSV were expressed as the reciprocal of the highest serum dilution in which no CPE was observed.

### rSPV-mp2 induced immune reactions

The immune response evoked by rSPV-mp2 was assessed indirectly by measuring the levels of IFN-γ and IL-4 in the serum. Each cytokine was detected using commercial ELISA kits (ExCell Bio, China) according to the manufacturer’s instructions.

### Gross pathology and histopathology examinations

Necropsy and gross pathological examinations of the lungs were immediately performed in the piglets that died during the experiment, or once the survived piglets were humanely euthanized at the termination of the experiment. Pigs were anesthetized and sacrificed by an intramuscular injection containing a combination of ketamine (20 mg/kg, China Animal Husbandry Group, China) and xylazine (2 mg/kg, China Animal Husbandry Group, China) followed by an intravenous injection of a pentobarbital sodium overdose (60 mg/kg, China Animal Husbandry Group, China). Macroscopic and microscopic lung lesions were observed and scored blindly as previously described^46^. The percentage of total lung containing macroscopic lesions was calculated using the following formula: 100 × [(0.10 × left anterior lobe) + (0.10 × left middle lobe) + (0.275 × left caudal lobe) + (0.10 × right anterior lobe) + (0.10 × right middle lobe) + (0.275 × right caudal lobe) + (0.05 × accessory lobe)]. For the microscopic lung lesions, an estimated score of the interstitial pneumonia severity was given on a scale ranging from 0 (normal) to 4 (severe interstitial pneumonia).

### Immunohistochemistry

Lung tissues were collected at necropsy for immunohistochemistry (IHC) as previous described^32^. A monoclonal antibody against PRRSV (mAb A2) was used as the primary antibody for detecting PRRSV-positive cells. To obtain quantitative data, the detection was executed through a ranked score between 0–4, which was used to evaluate the number of positive cells per section taken from each block. The indications for the scores were as follows: 0 = no positive cells; 1 = 1–10 positive cells; 2 = 11–30 positive cells; 3 = 31–1000 positive cells; and 4 =  or > 100 positive cells.

### Statistical analysis

The data are presented as the mean ± S.D. All data were analysed using a one-way ANOVA. Statistical analyses were performed using SPSS v.16. Values of *P* < 0.05 were considered statistically significant.

### Ethics

We confirmed that all experiments were performed in accordance with the relevant guidelines and regulations of the Science and Technology Agency of Jiangsu Province. The Nanjing Agricultural University Veterinary College academic board approved all experiments in this research.

## Additional Information

**How to cite this article:** Lin, H. *et al*. Construction and immunogenicity of a recombinant swinepox virus expressing a multi-epitope peptide for porcine reproductive and respiratory syndrome virus. *Sci. Rep.* **7**, 43990; doi: 10.1038/srep43990 (2017).

**Publisher's note:** Springer Nature remains neutral with regard to jurisdictional claims in published maps and institutional affiliations.

## Figures and Tables

**Table 1:** Phage enrichment after panning random peptide libraries with mAbs.

Round of Panning	A1			A2			A7				
	1	2	3	1	2	3	1	2	3		
Input	1.0 × 10^11^	1.1 × 10^11^	1.2 × 10^11^	1.0 × 10^11^	1.2 × 10^11^	1.3 × 10^11^	1.0 × 10^11^	1.5 × 10^11^	1.0 × 10^11^		
Output	7.8 × 10^3^	1.4 × 10^5^	1.5 × 10^6^	6.7 × 10^3^	3.7 × 10^5^	5.2 × 10^6^	1.3 × 10^5^	3.0 × 10^6^	1.8 × 10^7^		
Wash	2.1 × 10^4^	5.1 × 10^4^	1.6 × 10^3^	5.7 × 10^4^	8.4 × 10^4^	7.5 × 10^3^	7.0 × 10^5^	7.0 × 10^4^	1.4 × 10^4^		
Output/Input	7.8 × 10^−8^	1.3 × 10^−6^	1.3 × 10^−5^	6.7 × 10^−8^	3.1 × 10^−6^	4.0 × 10^−5^	1.3 × 10^−6^	2.0 × 10^−5^	1.8 × 10^−4^		
Wash/Output	269%	36.4%	0.12%	851%	22.7%	0.144%	538%	2.33%	0.078%		
Fold enrichment	1	17	167	1	46	597	1	15	138		

**Table 2:** Macroscopic and microscopic lung lesion score and immunohistochemical antigen score of PRRSV for each treatment group.

Group	n	Gross score	Microscopic score	IHC signal counting
rSPV-mp2	5	1.47 ± 0.33^a^	1.05 ± 0.19^a^	12.5 ± 4.6^a^
Inactive PRRSV	5	1.54 ± 0.45^a^	1.12 ± 0.26^a^	14.3 ± 3.5^a^
wtSPV	5	6.82 ± 0.50^b^	4.86 ± 0.54^b^	75.5 ± 6.5^b^
PBS	5	7.15 ± 0.34^b^	4.92 ± 0.47^b^	76.8 ± 7.1^b^
Empty control*	5	0.00 ± 0.00^c^	0.00 ± 0.00^c^	0.00 ± 0.00^c^

Different letters (a, b, c, and d) indicate significant difference (*P* < 0.05) between groups.

*Pigs in empty control group were not immunized and not challenged.

**Table 3:** The neutralizing titers of these three mAbs against homologous and heterogeneous PRRSV strains.

Neutralization titers	A1	A2	A7
JS07	1 × 2^13^	1 × 2^14^	1 × 2^14^
NADC30	1 × 2^10^	1 × 2^12^	1 × 2^11^
