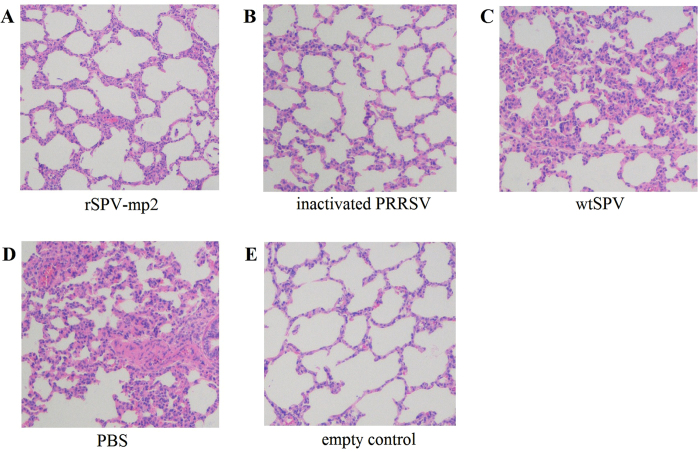# Corrigendum: Construction and immunogenicity of a recombinant swinepox virus expressing a multi-epitope peptide for porcine reproductive and respiratory syndrome virus

**DOI:** 10.1038/srep46592

**Published:** 2017-04-21

**Authors:** Huixing Lin, Zhe Ma, Xin Hou, Lei Chen, Hongjie Fan

Scientific Reports 7: Article number: 4399010.1038/srep43990; published online: 03 08 2017; updated: 04 21 2017

This Article contains errors in the order of Figures 1, 2, 4 and 5 which were inadvertently published as Figures 5, 4, 2 and 1 respectively. The correct Figures appear below. The legends for the Figures are correct.

## Figures and Tables

**Figure 1:**
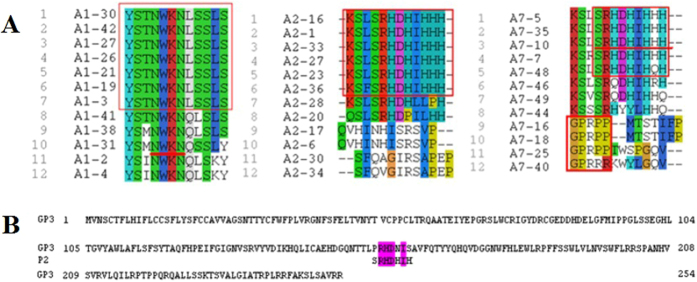


**Figure 2:**
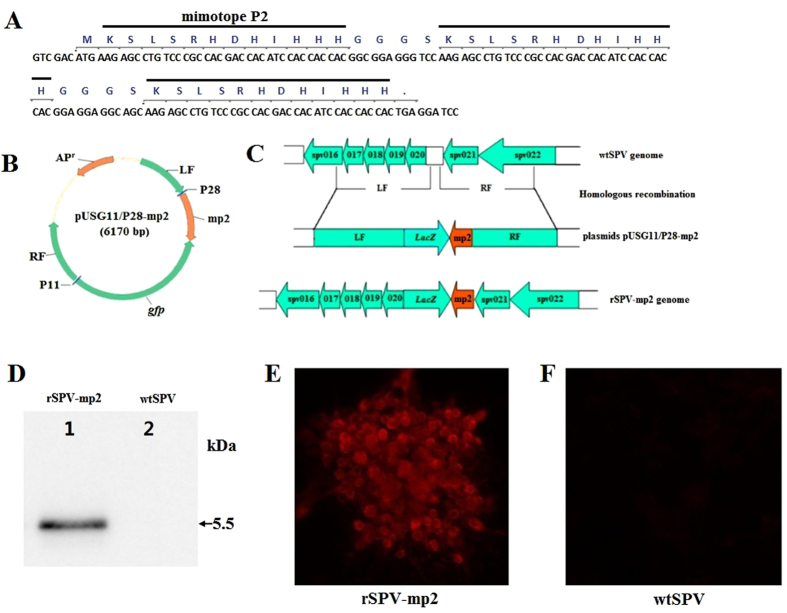


**Figure 4:**
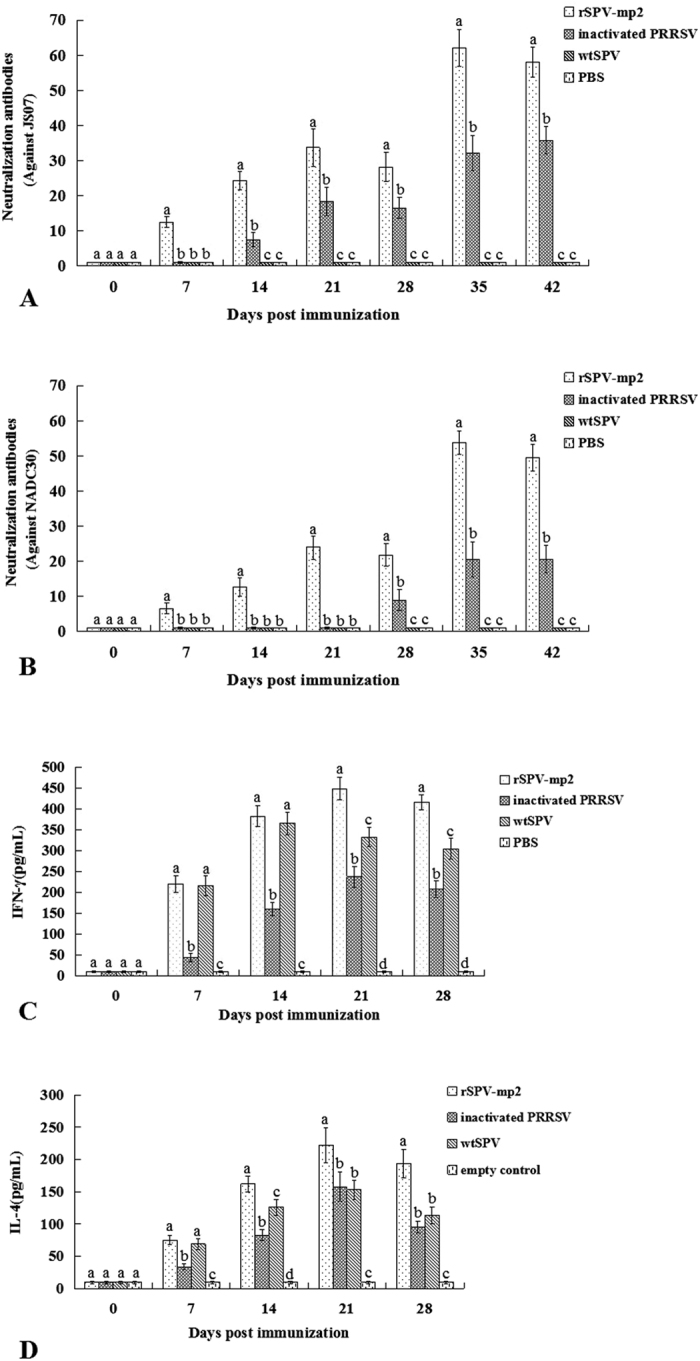


**Figure 5:**